# Lymphatic Programing and Specialization in Hybrid Vessels

**DOI:** 10.3389/fphys.2020.00114

**Published:** 2020-02-20

**Authors:** John B. Pawlak, Kathleen M. Caron

**Affiliations:** Department of Cell Biology and Physiology, The University of North Carolina at Chapel Hill, Chapel Hill, NC, United States

**Keywords:** lymphatic, endothelium, hybrid vessel, liver sinusoid, high endothelial venule

## Abstract

Building on a large body of existing blood vascular research, advances in lymphatic research have helped kindle broader investigations into vascular diversity and endothelial plasticity. While the endothelium of blood and lymphatic vessels can be distinguished by a variety of molecular markers, the endothelia of uniquely diverse vascular beds can possess distinctly heterogeneous or hybrid expression patterns. These expression patterns can then provide further insight on the development of these vessels and how they perform their specialized function. In this review we examine five highly specialized hybrid vessel beds that adopt partial lymphatic programing for their specialized vascular functions: the high endothelial venules of secondary lymphoid organs, the liver sinusoid, the Schlemm’s canal of the eye, the renal ascending vasa recta, and the remodeled placental spiral artery. We summarize the morphology and endothelial expression pattern of these vessels, compare them to each other, and interrogate their specialized functions within the broader blood and lymphatic vascular systems.

## Introduction

The discovery of new lymphatic markers has been a boon for lymphatic research helping to improve our understanding of the development and maintenance of lymphatics, as well as the identification of new lymphatic vessels. Historically, the distinction between blood and lymphatic vessels has been both categorical and demarcated. However, with new discoveries, we find that endothelial identity is more malleable and impressionable than previously thought. The combined expression of endothelial markers, lymphatic or otherwise, is often indicative of a specific set of functions conferred by the endothelium which possesses these markers (Potente and Makinen, 2017). For example, marker expression on lymphatic capillaries, blunt-ended vessels specialized to support trans-endothelial fluid and cellular transport, differs from that of collecting vessels, which are specialized to support intraluminal transport. The marker expression of these vessels further differ from valves, designed to block or allow intraluminal passage based on pressure gradients, and lymph node lymphatics which are more specialized for the presentation of antigens. Furthermore, the endothelia of these different lymphatic structures are influenced by signals from the local micro- and macro-environment, including from immune cells, extracellular matrix, and organ-specific cells and structures, which is most starkly exemplified by the changes that occur during tumor-associated lymphatic remodeling (Lutter and Makinen, 2014; Petrova and Koh, 2018; Wong et al., 2018; Garnier et al., 2019).

With these discoveries, it has become clear that the endothelium is far more plastic than previously thought and that transitions between blood and lymphatic identities occur (Ma and Oliver, 2017; Azimi et al., 2019). As such, a growing number of endothelial lined vessels which do not fit neatly into a classical vessel type have been identified. Recently, three different vessels, termed hybrid vessels, have been found to possess a combination of blood and lymphatics markers, presumably necessary for their highly specialized function: the Schlemm’s canal (SC) of the eye, the ascending vasa recta (AVR) of the renal medulla, and the remodeled spiral arteries (rSA) of the placental decidua (Aspelund et al., 2014; Park et al., 2014; Truong et al., 2014; Kenig-Kozlovsky et al., 2018; Pawlak et al., 2019). These vessels are in addition to an existing small catalog of highly specialized non-lymphatic vessels with similarly distinct expression patterns, including the liver sinusoid and high endothelial venules (HEV) of secondary lymphoid organs. In this review we compare and contrast the unique functions and expressional programing of these vessels to each other and to the classical features of blood and lymphatic vasculature and discuss the implications of these hybrid vessels within the greater context of physiology and endothelial biology.

## Blood Vasculature

There are three fundamental blood vessel categories within the body: arteries carry blood away from the heart, capillaries exchange gasses and nutrients with local tissue, and veins transport blood back to the heart. Additionally, collateral vessels can shunt blood between vascular beds. Blood vessels are composed of three distinct layers (Mazurek et al., 2017). The tunica intima is the inner most layer with a single layer of endothelial cells (EC), basement membrane, and connective tissue. The endothelium and basement membrane in this layer can morphologically vary to allow for more or less permeability; continuous endothelia and basement membranes are less permeable than discontinuous ones with gaps allowing for passage of larger materials. The middle layer, called tunica media, is thicker in arteries than in veins and contains smooth muscle cells with connective and elastic tissue. The outer tunica adventitia layer, which is often thicker in veins than in arteries, anchors the vessel to the local tissue with elastic and connective fibers. The endothelium of capillaries instead have no smooth muscle, but sometimes have a discontinuous layer of pericytes. The permeability of capillaries vary based on location, but have universally small lumens which allows for gas and nutrient exchange between blood and local tissue.

A variety of versatile endothelial markers exist for the examination of the blood vasculature, including the tyrosine kinase receptors VEGFR1 and TIE2, the blood plasma and Weibel-Palade bodies-bound glycoprotein vWF, and the membrane glycoprotein PLVAP (Jaffe et al., 1973; Sato et al., 1995; Ferrara and Davis-Smyth, 1997; Yamamoto et al., 1998; Stan et al., 1999a, b). Additionally, the platelet endothelial adhesion molecule CD31, and the phosphoglycoprotein CD34, are often used as blood-specific endothelial markers, but can also be weakly expressed on lymphatic endothelium (van Mourik et al., 1985; Kriehuber et al., 2001; Podgrabinska et al., 2002). Distinct markers can differentiate between arterial and venous endothelium. In particular, the receptor tyrosine kinase EphB4 is a venous marker, while its cognate membrane-bound ligand ephrin B2 marks arterial endothelium, though both are expressed in blood capillaries (Wang et al., 1998). Additionally, the VEGFR2 co-receptors NRP1 and NRP2 are differentially expressed, the former is expressed on arterial endothelium and the latter on venous endothelium (Herzog et al., 2001). However, NRP2 is also a co-receptor of VEGFR3 on lymphatic endothelium, making it a more promiscuous venous marker (Yuan et al., 2002). Similarly, the mucin-like sialoglycoprotein, endomucin, preferentially expresses on both venous and lymphatic endothelia (Samulowitz et al., 2002). Pan-endothelial markers are widely used and valuable based on context and application. Commonly used pan-endothelial markers include the tyrosine kinase receptor VEGFR2, and the endothelial junctional protein, VE-Cadherin (Lampugnani et al., 1992; Shalaby et al., 1995; Dellinger et al., 2013; Hagerling et al., 2018).

## Lymphatic Vasculature

Lymphatic vessels are responsible for maintaining fluid homeostasis, fat absorption, and immune cell trafficking. Excess interstitial fluid drains as lymph into blind-ended lymphatic capillaries that contain a permeable, discontinuous basement membrane and overlapping ECs that collectively function like unidirectional valves (Baluk et al., 2007). Lymph is then transported into lymphatic collectors, which have a structure similar to veins. Collectors have a basement membrane, lymphatic muscle cells, pericytes, and are regularly segmented by one-way endothelial valves (Oliver, 2004). Lymph can be pushed though the one-way valves by external pressures and phasic contractile forces generated by collector lymphatic muscle cells, effectively facilitating basal to apical flow (Muthuchamy et al., 2003). Collecting vessels can then move lymph through lymph nodes for antigen presentation to immune cells, and then drain back into the venous circulation (Randolph et al., 2017).

Formation of the mouse lymphatic vasculature begins around embryonic day 9.5 when a subpopulation of ECs on the cardinal vein express PROX1, a master regulator of lymphatic fate, which requires expression of the transcription factors SOX18 and COUP-TFII, the latter of which is considered a master regulator of venous fate (Wigle and Oliver, 1999; You et al., 2005; Francois et al., 2008). The PROX1^+^ cardinal vein ECs, which also express the membrane glycoprotein LYVE1, bud off the vein and acquire the expression of another membrane glycoprotein, podoplanin (PDPN) (Banerji et al., 1999; Breiteneder-Geleff et al., 1999). The ECs coordinately form a primitive lymph sac at embryonic day 11.5 in a process primarily mediated by VEGFR3/NRP2/VEGFC signaling (Karkkainen et al., 2004). Lymphangiogenesis from the lymph sac then forms the peripheral lymphatic network, supported by endothelial proliferation promoted by AM/CLR/RAMP2 signaling (Fritz-Six et al., 2008). Lymphatic valves are formed to help promote unidirectional flow toward the venous circulation, while a lymphovenous valve prevents blood from flowing into the lymphatics (El Zawahry et al., 1983). Notably, both types of valve LECs have distinct expression patterns from each other and more so from vessel wall LECs (Scallan et al., 2016; Janardhan and Trivedi, 2019).

Through derivation from the primitive lymph sac, most of the lymphatic vasculature derives from a venous origin (Srinivasan et al., 2007; Yang et al., 2012; Hagerling et al., 2013; Escobedo and Oliver, 2016). However, newer evidence in mice suggests that a subset of LECs are derived from non-venous sources in the heart (Klotz et al., 2015), dermis (Martinez-Corral et al., 2015), and mesentery (Mahadevan et al., 2014; Stanczuk et al., 2015), as well as LECs derived from the mesenchyme (Buttler et al., 2006; Wilting et al., 2006; Ulvmar and Makinen, 2016). Non-venous LECs were primarily identified by lineage tracing experiments with the venous/endothelial cell marker TIE2. Evidence suggests that LECs lacking TIE2 labeling in the heart and mesentery may instead derive from a hemogenic endothelium population, whereas the origin of non-venous dermal LECs currently remains unknown (Mahadevan et al., 2014; Klotz et al., 2015; Stanczuk et al., 2015).

## High Endothelial Venules

**FIGURE 1:**
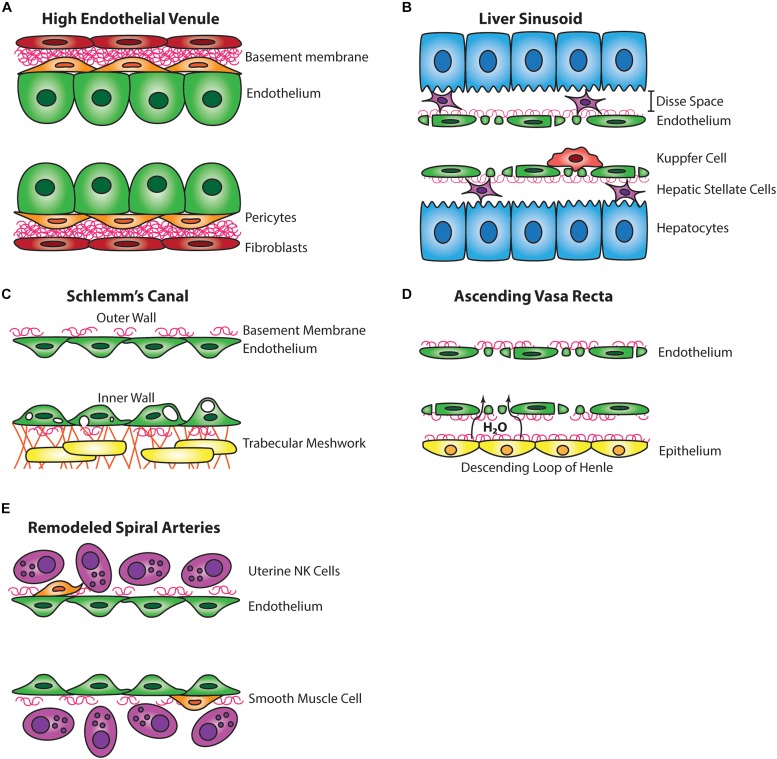
Comparative wall structure of hybrid vessels. **(A)** The endothelial cells of high endothelial venules are tall and cuboidal and are associated with pericytes and a thick basement membrane. **(B)** The endothelium of the liver sinusoid is fenestrated, lacks mural cells, is supported by a thin basement membrane, and is associated with intraluminal Kupffer cells. The Disse space separates the liver sinusoid endothelium from hepatocytes and is inhabited by hepatic stellate cells. **(C)** The Schlemm’s canal has a discontinuous basement membrane, lacks mural cells, and the inner wall endothelium forms large vacuoles in response to aqueous humor flow from the trabecular meshwork. **(D)** The endothelium of the ascending vasa recta is fenestrated, lacks mural cells, and takes up water from the descending loop of Henle. **(E)** The endothelium of the remodeled spiral artery has minimal smooth muscle coverage, a discontinuous basement membrane, and closely associates with uterine NK cells.

The HEV are post-capillary swellings of venous blood vessels that are especially adapted for trafficking of lymphocytes. HEVs are found in secondary lymphoid organs, including lymph nodes and the Peyer’s patch of the small intestine, and are required for the function and organogenesis of these organs by recruiting essential lymphocyte populations. HEVs are covered by overlapping pericytes in a thick basement membrane, and HEV ECs are distinct from other blood ECs by their thick (or high) cuboidal shape, from which the vessel’s name derives (Figure 1A; Ager, 2017).

While the expression pattern of HEV ECs can have some variability by organ, HEV ECs have generally been found to express the lymphatic markers VEGFR3, LYVE1, and CCL21, but not PROX1 (Table 1; Gunn et al., 1998; Lacorre et al., 2004; Wrobel et al., 2005). LYVE1 functions as receptor for hyaluronan which is metabolized in the lymph node (Fraser and Laurent, 1989). CCL21, a ligand for CCR7, functions as a chemoattractant for immune cells (Yoshida et al., 1998). VEGFR3 expression on HEVs is controversial, but an abundance of evidence supports that it is expressed in many studies (Lacorre et al., 2004; Farnsworth et al., 2011). HEV ECs also express endothelial markers CD31, CD34, VE-Cadherin, and VEGFR2, and the blood endothelial markers vWF, PLVAP, and VEGFR1 (Pfeiffer et al., 2008; Farnsworth et al., 2011). As found on other venules, HEVs also express the membrane-bound glycoprotein endomucin, where it likely functions as a regulator of cellular adhesion (Samulowitz et al., 2002). Interestingly, HEV ECs do not express PDPN, but fibroblastic reticular cells that surround the HEV do and that expression is required to maintain VE-Cadherin expression and barrier function of HEV ECs (Farr et al., 1992; Herzog et al., 2013).

**TABLE 1:** Expression pattern of hybrid vessels.

Endothelial marker	Common endothelial expression				Schlemm’s Canal		Ascending vasa recta		Remodeled spiral arteries		High endothelial venules		Liver sinusoid	
PROX1	L			Wigle and Oliver, 1999	+	Aspelund et al., 2014; Park et al., 2014; Truong et al., 2014	+	Kenig-Kozlovsky et al., 2018	+	Pawlak et al., 2019	−	Lacorre et al., 2004	−	Mouta Carreira et al., 2001
LYVE1	L			Banerji et al., 1999	−	Aspelund et al., 2014; Park et al., 2014; Truong et al., 2014	−	Kenig-Kozlovsky et al., 2018	±	Pawlak et al., 2019	+	Liao and Ruddle, 2006	+	Mouta Carreira et al., 2001
PDPN	L			Breiteneder-Geleff et al., 1999	−	Aspelund et al., 2014; Park et al., 2014	−	Kenig-Kozlovsky et al., 2018	−	Pawlak et al., 2019	−	Herzog et al., 2013	±^3^	Yokomori et al., 2010
CCL21	L			Kriehuber et al., 2001	+	Aspelund et al., 2014	NRF		−	Pawlak et al., 2019	+	Gunn et al., 1998	−	Grant et al., 2002
Integrin α9	L			Huang et al., 2000; Bazigou et al., 2009	+	Aspelund et al., 2014; Park et al., 2014	NRF		NRF		NRF		+	Couvelard et al., 1998
VEGFR3	L	|V|	|A|	Kaipainen et al., 1995	+	Aspelund et al., 2014; Park et al., 2014	+	Kenig-Kozlovsky et al., 2018	+	Pawlak et al., 2019	±	Lacorre et al., 2004; Farnsworth et al., 2011	+	Yamane et al., 1994
SOX18	L	|V|	|A|	Pennisi et al., 2000; Francois et al., 2008	±	Park et al., 2014	NRF		NRF		NRF		+	Matsui et al., 2006
NRP2	L	V		Herzog et al., 2001; Yuan et al., 2002	NRF		NRF		−	Germeyer et al., 2005; Pawlak et al., 2019	NRF		−	Elpek, 2015
Endomucin	L	V		Samulowitz et al., 2002	+	Park et al., 2014	+	Kenig-Kozlovsky et al., 2018	−	Pawlak et al., 2019	+	Samulowitz et al., 2002	+	Walter et al., 2014
CALCRL	L	V	A	Nagaya et al., 2005; Fritz-Six et al., 2008	NRF		NRF		+	Li et al., 2013	NRF		+	MacParland et al., 2018
KLF4	L	V	A	Yet et al., 1998; Dieterich et al., 2015	+	Park et al., 2014	NRF		NRF		NRF		NRF	
VE-Cadherin	L	V	A	Lampugnani et al., 1992; Hagerling et al., 2018	+	Perkumas and Stamer, 2012; Kizhatil et al., 2014	NRF		+	Bulla et al., 2005	+	Pfeiffer et al., 2008	±	Lalor et al., 2006; Ding et al., 2010
VEGFR2	L	V	A	Shalaby et al., 1995; Dellinger et al., 2013	+	Perkumas and Stamer, 2012; Kizhatil et al., 2014	−	Kenig-Kozlovsky et al., 2018	NRF^1^	Hirashima et al., 2003	+	Farnsworth et al., 2011	+	Yamane et al., 1994
FOXC2	L	|V|	A	Dagenais et al., 2004; Seo et al., 2006	+	Aspelund et al., 2014; Park et al., 2014	NRF		NRF		NRF		NRF	
EPHB4		V		Wang et al., 1998	NRF		NRF		+	Zhang et al., 2008	NRF		+	Das et al., 2010
CD31	*l*	V	A	van Mourik et al., 1985; Podgrabinska et al., 2002	+	Aspelund et al., 2014; Park et al., 2014	+	Kenig-Kozlovsky et al., 2018	+	Bulla et al., 2005; Pawlak et al., 2019	+	Pfeiffer et al., 2008	±	Lalor et al., 2006
CD34	*l*	V	A	Kriehuber et al., 2001; Podgrabinska et al., 2002	+	Kizhatil et al., 2014	+	Kenig-Kozlovsky et al., 2018	NRF		+	Wrobel et al., 2005	−	Lalor et al., 2006
TIE2	|*l*|	V	A	Sato et al., 1995; Shen et al., 2014	+	Perkumas and Stamer, 2012; Kizhatil et al., 2014	+	Kenig-Kozlovsky et al., 2018	+	Goldman-Wohl et al., 2000	+	Hayasaka et al., 2010	+	Poisson et al., 2017
PLVAP		V	A	Stan et al., 1999a, b	+	Herrnberger et al., 2012	+	Pannabecker and Dantzler, 2006; Kenig-Kozlovsky et al., 2018	NRF		+	Rantakari et al., 2015	+	Rantakari et al., 2016
vWF		V	A	Jaffe et al., 1973; Yamamoto et al., 1998	+	Hamanaka et al., 1992; Park et al., 2014	NRF^2^	Pupilli et al., 1997	+	Bulla et al., 2005	+	Lacorre et al., 2004	+	Lalor et al., 2006
VEGFR1		V	A	Ferrara and Davis-Smyth, 1997	+	Perkumas and Stamer, 2012; Fujimoto et al., 2016	+	Young et al., 2018	+	Hirashima et al., 2003	+	Hayasaka et al., 2010	−	Kato et al., 2011
Ephrin B2			A	Wang et al., 1998	+	Kizhatil et al., 2014	NRF		+	Zhang et al., 2008	NRF		+	Das et al., 2010; Mimche et al., 2018
NRP1			A	Herzog et al., 2001	+	Perkumas and Stamer, 2012	NRF		+	Germeyer et al., 2005	−	Lee et al., 2014	+	Elpek, 2015

*L = Lymphatic; A = Arterial; V = Venous; |L| |V| |A| = Transiently expressed during development; l v a = Weakly expressed; (+) = Expressed; (−) = Not expressed; (±) = Transiently or Discontinuously Expressed; NRF = No Reporting Found; (±) = Reported both as expressed and not expressed. ^1^Hirashima et al. (2003) report that VEGFR2 is not expressed in the SA of the proximal decidua at embryonic day 12.5, but only after the endothelium has been replaced by fetal endovascular trophoblasts. Their data appears to show LacZ reporter staining for VEGFR2 in endothelial lined SAs, but this was not explicitly reported by the authors (Hirashima et al., 2003). This important distinction has not been made in some previous review articles (Rai and Cross, 2014). ^2^Staining done by Pupilli et al. (1997) shows low or absent vWF expression in ascending vasa recta, while the descending vasa recta shows clear expression, but the ascending vasa recta vWF staining was not explicitly reported by the authors. ^3^Yokomori et al. (2010) described PDPN as “scantly” expressed on LSECs. However, PDPN expression has been described on Kupffer cells which localized to the sinusoids which was not addressed in the study by Yokomori et al. (2010) and Hitchcock et al. (2015).*

## Liver Sinusoid

The liver sinusoid is a network of capillaries lined by a discontinuous endothelium. Liver sinusoidal ECs (LSECs) are flat and highly fenestrated (covering ∼20% of the surface), allowing for passage of plasma across the endothelium into the space of Disse located between the endothelium and adjacent hepatocytes (Figure 1B). The passage of plasma across LSECs is important for hepatic blood clearance of harmful compounds and drugs from the circulation. This clearance is supported by Kupffer cells within the sinusoid that phagocytose particles too large to pass through LSECs. LSECs have minimal basement membrane with a lack of an organized basal lamina and also lack tight junctions (Wisse, 1972).

LSECs have an interesting expression pattern that includes the lymphatic markers LYVE1, VEGFR3, and integrin α9, but not PROX1 (Table 1; Couvelard et al., 1998; Mouta Carreira et al., 2001). Similar to the lymph nodes, the liver metabolizes hyaluronan which its receptor, LYVE1, in LECS likely supports (Vrochides et al., 1996). PDPN has been described as “scantly” expressed on LSECs (Yokomori et al., 2010), but that study did not address if the signal came from PDPN-positive Kupffer cells within the sinusoid (Hitchcock et al., 2015). The pan-endothelial markers TIE2 and VEGFR2 are expressed on LSECs, however, the expression of some other pan-endothelial and blood markers in LSECs are unusually low (vWF, CD31) or absent (VE-Cadherin, CD34) (Lalor et al., 2006). Though it should be noted that the expressions of CD31 and VE-Cadherin are controversial since both have been reported to be either expressed or not expressed on LSECs (Lalor et al., 2006; Ding et al., 2010). The arterial and venous markers Ephrin B2 and EPHB4, respectively, are both expressed in LSECs, which is consistent with function of the liver sinusoid as a capillary plexus since Ephrin B2 and EPHB4 coordinately direct remodeling of capillary networks.

## Schlemm’s Canal

The SC of the eye is a channel encircling the periphery of the cornea with a continuous EC monolayer on a discontinuous basement membrane which facilitates bloodless basal-to-apical flow similar to lymphatic capillaries (Figure 1C; Ramos et al., 2007; Aspelund et al., 2014). The SC functions as a regulator of intraocular pressure by providing passage for aqueous humor from the trabecular meshwork, draining then into the aqueous and episcleral veins (Tamm, 2009). The aqueous humor outflow is similar between mice and humans. However, only about 20% of total aqueous humor outflow passes through the SC in mice (Aihara et al., 2003), whereas a majority of outflow passes through the SC in humans. Historically, the SC was thought to be a blood vessel due to the expression of blood endothelial markers, including vWF (Dautriche et al., 2015), but more recently the rodent SC has been noted to possess many characteristics of lymphatic vessels.

In 2014, three groups independently identified the expression of PROX1, a transcription factor master regulator of lymphatic fate, in the mouse SC (Aspelund et al., 2014; Park et al., 2014; Truong et al., 2014), and has since been identified in the SC of rats (Jung et al., 2017). Interestingly, only a subset of lymphatic markers are expressed in the SC, including VEGFR3, CCL21, integrin α9, and low or transient expression of SOX18 and FOXC2. However, LYVE1 and PDPN expression is not detected in SC ECs. Meanwhile, the blood endothelial markers CD31, vWF, and endomucin are expressed in the SC, as wells as the pan-endothelial markers VE-Cadherin and TIE2, suggesting that these vessels acquire a blood/lymphatic hybrid identity (Table 1; Aspelund et al., 2014; Park et al., 2014; Truong et al., 2014). This hybrid identity is hypothesized to help the SC to perform its highly specialized function in a way that neither a classic blood nor lymphatic vessel could perform. Indeed, VEGFR3 signaling is required for SC development (Aspelund et al., 2014), and recent work shows that PROX1 is required for development and maintenance of the SC. Also the blood vessel marker VEGFR2 is required for development but gradually decreases expression from p4 to adulthood during a simultaneous increase in PROX1 and VEGFR3 expression. Interestingly, the lymphatic programing of the SC is flow-mediated; PROX1 and VEGFR3 expression is significantly decreased when aqueous humor outflow is decreased (Park et al., 2014). Since humans rely more on SC-mediated outflow than mice, these findings may have increased relevance to the pathophysiology of the human eye.

More recent work shows that TIE2 (*Tek*) signaling is required for both development and maintenance of the SC, and is otherwise necessary to perform its drainage function (Thomson et al., 2014; Kim et al., 2017). During development, TIE2 is an important regulator of vascular remodeling and stability. In the SC and lymphatics vessels, ANGPT1 and ANGPT2 function as agonists of TIE2, which differs from blood vessels where ANGPT2 acts as an antagonist to ANGPT1 activation of TIE2 (Augustin et al., 2009). Agonist signaling of TIE2 is required for SC development; mice lacking ANGPT1 and ANGPT2 or TIE2 develop glaucoma due to an unformed SC, restricting aqueous humor outflow. Furthermore, inducible deletion of *Angpt1* and *Angpt2* or *Tek* in adult mice demonstrate that agonist TIE2 signaling is also required to maintain SC integrity and PROX1 expression (Kim et al., 2017).

## Ascending Vasa Recta

The AVR and descending vasa recta (DVR) of the renal inner medulla are blood vessels utilized for concentrating urine along the nephron (Pallone et al., 2003). The AVR in particular is highly fenestrated, lacks mural cell coverage, has a discontinuous basement membrane, and is important for fluid reabsorption back to the vasculature (Figure 1D; Schwartz et al., 1976; Takahashi-Iwanaga, 1991). These features are also attributed to lymphatic vessels which are otherwise absent or rare in the renal medulla (Russell et al., 2019). Similar to SC, the AVR expresses both PROX1 and VEGFR3, but not LYVE1 and PDPN. Additionally, the AVR expresses endomucin and blood endothelial markers CD31, CD34, VEGFR1, and PLVAP (Table 1; Kenig-Kozlovsky et al., 2018). Interestingly, despite typically being expressed in blood vessels, VEGFR2 expression was not found in the AVR (Kenig-Kozlovsky et al., 2018). VEGFR2 and VEGFR3 are able to hetero-dimerize and activate downstream signaling pathways, such as AKT signaling, that differ from those triggered by homo-dimerized VEGFR3, such as ERK signaling (Deng et al., 2015). Consequently, absence of VEGFR2 in these vessels could lead to increased VEGFR3 homo-dimer signaling (Dixelius et al., 2003). As postulated by Kenig-Kozlovsky et al. (2018) VEGFR3 may be implicated in vessel widening of the AVR, which is significantly wider than the DVR, as VEGFR3 responds to fluid shear stress by promoting outward vessel remodeling (Baeyens et al., 2015).

To date, the developmental origin of the AVR is not well understood. Further, it is unclear whether the AVR derives from a unique set of progenitor cells or undergoes differentiation while under development. It is likely that the acquisition of VEGFR3 is due in large part to PROX1 transcriptional activity (Hong et al., 2002; Petrova et al., 2002), but it is unclear what initiates PROX1 expression in the AVR. Examination of known promoters of PROX1 expression, such as SOX18 and KLF4, may help identify early regulatory mechanisms that push the AVR toward a hybrid identity.

The AVR also expresses TIE2 which is required for development of the AVR, but not the DVR (Kenig-Kozlovsky et al., 2018). Constitutive deletion of *Tek* or simultaneous deletion of *Angpt1* and *Angpt2* leads to early embryonic lethality (Dumont et al., 1994; Sato et al., 1995), but Kenig-Kozlovsky et al. (2018) overcame this lethal phenotype by inducing deletion of *Tek* or *Angpt1* and *Angpt2* simultaneously at embryonic day 16.5. These mice develop renal cysts and have reduced urine concentrating ability which is attributed to the absence of the AVR, despite the normal formation of the DVR (Kenig-Kozlovsky et al., 2018).

## Spiral Arteries

The spiral arteries (SA) of the decidual placenta are tortuous maternal blood vessels that transport maternal blood to the fetal side of the placenta where gasses and nutrients are exchanged with the fetal vasculature. With the support of local uterine NK cells, SAs must remodel during midgestation to increase blood flow and nutrient delivery to support the growing fetus (Moll et al., 1978). In humans, poor SA remodeling is associated with pregnancy complications, including fetal growth restriction, preterm birth, and preeclampsia (Lyall, 2002; Pijnenborg et al., 2006), and can otherwise lead to long-term health complications for mother and child (Barker et al., 1989; Gastrich et al., 2010; Geelhoed and Jaddoe, 2010). Prior to remodeling, the SA is supported by smooth muscle coverage, extracellular matrix, and a basement membrane, but during SA remodeling smooth muscle coverage is shed, extracellular matrix degrades, and basement membrane is diminished (Figure 1E; Sweeney et al., 2006; Whitley and Cartwright, 2010; Robson et al., 2012). These changes help facilitate an increase in lumen diameter and reduced tortuosity to promote increased blood flow.

Our group recently discovered that rodent SAs acquire expression of a subset of lymphatic markers, similar to the SC and AVR (Pawlak et al., 2019). We found that the SA acquires these lymphatic markers during the remodeling period between embryonic day 11.5 to embryonic day 13.5 when smooth muscle coverage is shed and the lumen diameter expands. Similar to the SC and AVR, the remodeled SA (rSA) expresses PROX1 and VEGFR3, but not PDPN, as well as TIE2 and CD31 (Table 1; Goldman-Wohl et al., 2000). VEGFR3 expression in particular persists throughout pregnancy. Our work suggests that the rSA utilizes lymphatic expression to help expand the lumen diameter and modify the vascular tone to allow for increased blood flow, and that VEGFR3/VEGFC signaling in particular plays a central role in that function. The expression of VEGFR3 allows the SA to become responsive to locally secreted VEGFC which is required to promote remodeling. However, unlike the aforementioned hybrid vessels, the rSA expresses LYVE1, but only in a non-continuous subset of ECs. Also unlike the SC, the rSA does not express CCL21, which may be related to the immune privileged nature of the placenta intended to prevent an immunological maternal response to fetal antigens (Weber et al., 2013). Additionally, the rSA is not a fenestrated vessel and does not express endomucin, which is often associated with vessels that help perform fluid homeostasis.

Fundamentally, the differences in expression in the rSA compared to the other hybrid vessels likely relates to the physiological differences in function and origin of these vessels. The rSA regulates blood transport, the SC regulates aqueous humor fluid homeostasis, and the AVR serves as both a transport and homeostasis vessel. Though one key difference between rSAs and the other hybrid vessels and lymphatics is that rSAs are arterial while the others are of a venous origin (Srinivasan et al., 2007). It is important to note that some LECs are derived from a non-venous origin (Ulvmar and Makinen, 2016), and while VEGFR3 is regarded as a lymphatic marker in adult vessels, it is also expressed in fetal blood vessels, including arterioles. Furthermore, it should be appreciated that SAs acquire transient expression of the venous blood vessel marker EPHB4 prior to lymphatic expression, suggesting a shift from arterial to venous to lymphatic identity that may help facilitate the full transition and remodeling process (Zhang et al., 2008).

## Closing Remarks

The molecular profile of hybrid vessels is related to the unique microenvironments in which they are located and the highly specialized functions they perform, and cell-autonomous mechanisms may also contribute to their heterogeneity. Notably, the molecular profiles of mature hybrid vessels appear to be plastic, as evident by expressional changes to HEVs in response to immunization (Liao and Ruddle, 2006). Indeed, this vessel plasticity under aberrant signaling is presumed to contribute to certain pathologies, such as poor spiral artery remodeling in preeclampsia and poor aqueous humor drainage via the SC in glaucoma (Karpinich and Caron, 2014; Pawlak et al., 2019).

It is worth commenting that some of the newly characterized hybrid vessels are found in regions that are either currently or historically believed to be devoid of lymphatic vessels (Dickinson and Gausas, 2006; Red-Horse et al., 2006; Castro et al., 2011; Lee et al., 2011). Certainly, the expansion of diverse and reliable lymphatic markers has enabled more precise characterization of these structures. It is also evident that the field is moving away from overreliance on only one or two lymphatic markers in *in vivo* studies, not only because expression patterns on vessels may be altered by local or organ-specific factors, but also because no single marker identified to date is completely exclusive to lymphatic vessels. Finally, instances of programmed and pathological endothelial mimicry could further complicate molecular profiling, particularly in a tumor environment.

It is likely that more uncharacterized hybrid vessels still remain to be identified. As with the aforementioned hybrid vessels, vascular beds with highly specialized functions may be ripe for the examination of lymphatic markers which may have been coopted to promote the specialized morphology that serves their function. Indeed, understanding how the lymphatic vasculature responds to pathological conditions may be informative in the identification and characterization of potential hybrid vessels (see the comprehensive review on this topic by Padera et al. (2016). Ultimately, what qualifies as a hybrid vessel may be debatable or evolve over time. Nevertheless, this can be considered a natural and positive outcome of our expanding appreciation for the breadth of endothelial plasticity and heterogeneity.

## Author Contributions

JP researched the literature and wrote the manuscript. KC provided direction on, reviewed, and edited the manuscript.

## Conflict of Interest

The authors declare that the research was conducted in the absence of any commercial or financial relationships that could be construed as a potential conflict of interest.
